# Practitioner and Service User Perspectives on the Rapid Shift to Teletherapy for Individuals on the Autism Spectrum as a Result of COVID-19

**DOI:** 10.3390/ijerph182211812

**Published:** 2021-11-11

**Authors:** Genevieve Johnsson, Kim Bulkeley

**Affiliations:** 1 Individual and Community Services, Autism Spectrum Australia, Sydney 2153, Australia; 2 Centre for Disability Research and Policy, University of Sydney, Sydney 2153, Australia; kim.bulkeley@sydney.edu.au

**Keywords:** teletherapy, telehealth, telepractice, disability, autism, allied health, COVID, allied health professionals

## Abstract

Prior to COVID-19, research into teletherapy models for individuals on the autism spectrum was slowly progressing. Following the onset of COVID-19, teletherapy became a necessity for continuity of services, however, research was still emerging for how to translate best practice autism support to the online environment. The aim of this research was to gain insight into the rapid shift to teletherapy for practitioner and service users and the implications for the broader disability sector. Survey responses were collected from 141 allied health practitioners (speech pathologists, occupational therapists, psychologists, educators, and social workers) from four Australian states and territories. A total of 806 responses were collected from service users following an individual teletherapy session. Five themes were identified during the qualitative analysis; (1) technology—love it or hate it; (2) teletherapy as a “new normal”; (3) short term pain, for long term gain; (4) the shape of service delivery has changed; (5) is teletherapy always an option? Data from the quantitative analysis provided further insights into the first two themes. While COVID-19 has brought forward significant advances in telehealth models of practice, what is needed now is to delve further into what works, for who, and in which context, and explore the potentiality, efficiencies, and scalability of a post-pandemic hybrid approach. This will inform practice guidelines and training, as well as information for service users on what to expect.

## 1. Introduction

Following the onset of COVID-19 in Australia in March 2020 when public health orders were implemented to reduce social contacts, a lack of access to in person face to face therapeutic support services impacted people on the autism spectrum. In geographically isolated regions and urban areas, teletherapy became not just a choice but a necessity for the continuity of services [1]. Teletherapy incorporates the use of telecommunications such as telephone, email and video conferencing to deliver therapeutic supports to individuals at a distance from the therapy provider [2]. The National Disability Insurance Scheme (NDIS) in Australia [3] operates on a reimbursement model that has supported telepractice, however this option has not been taken up extensively within the scheme. Prior to COVID-19, research into teletherapy models of practice for individuals on the autism spectrum in geographically isolated regions was emerging, with promising findings of benefits for children with autism and their families. In a systematic review in 2010, eight studies reported favourable outcomes [4] and, similarly, 14 studies in 2018 were positive about the impact of teletherapy [5]. However, both noted the lack of research on direct interventions or assessment provided by clinicians with children and young people with autism.

Teletherapy, as part of the broader terms of telehealth and telepractice, has been used successfully for a range of autism-specific interventions, including speech and language development [6], behaviour support [7,8,9,10], parent-mediated social-communication interventions for young child [11,12,13], and classroom coaching for educators of autistic students [14]. While reviews of the literature on teletherapy have suggested that the platform itself has not been a barrier for successful outcomes [15], the research is still emerging on how practitioners can translate best practice autism support to the online environment [5]. In a pilot study of multidisciplinary teletherapy services, Johnsson, et al. [16] found that some areas of practice were perceived by practitioners as being more difficult to adapt to online delivery, for example, fine and gross motor goals.

Many of the more recent studies on teletherapy have built on past research training of parents and practitioners in the implementation of Applied Behaviour Analysis for children on the autism spectrum [17,18,19,20,21], with positive findings supporting the inclusion of teletherapy as part of service delivery. These studies, however, have little qualitative data exploring the participant and practitioner perspectives of this adapted model of practice to flesh out the experience of teletherapy.

In a recent study of an occupational therapy teletherapy intervention for children on the autism spectrum, Wallisch et al. [22] found that as well as the bonus of less travel and increased access to supports, parents also learned to problem solve new situations and had the time to reflect on situations with the occupational therapist and gain confidence in trying new strategies. One of the key benefits reported by parents was being able to place therapy in the contexts, routines, and situations of the natural family environment, rather than the clinic [22]. Benefits in travel and family involvement when comparing remote and face to face early intervention programs have been found, however, parents, remote therapists and local support team members have highlighted the value of initial face to face support [23]. Conversely, others report remote delivery was reported to be limited in connecting the therapist to the child’s local context and capture non-verbal communication [24].

The observation of client and parent behaviour is an essential component of effective teletherapy services, and without proper set up and management by the practitioner, may be a barrier for the delivery of good quality services [25]. Practitioners require significant training and support to adapt their practice to a teletherapy model, and this had implications in a practitioner’s willingness to adopt this model of practice to achieve quality outcomes and role satisfaction [16].

Recent preliminary results from research conducted during the COVID-19 lockdowns [26] indicated that the loss of in person face to face support for adults on the spectrum has significantly impacted their mental health, and families have struggled to support their children to engage with their therapist via teletherapy. Another COVID-19 study from the United States [27] found that the use of coaching methods as part of telepractice for families of children with Autism Spectrum Disorder had positive outcomes, improving daily living skills. Given the inconsistencies in the literature on the efficacy of a teletherapy model for supporting individuals on the autism spectrum, more research is needed to understand practice in an online environment from those who have had this experience. Following the rapid shift to online service delivery, primarily offered as video conferencing therapy sessions, in late March 2020 due to COVID-19 public health recommendations, Autism Spectrum Australia (Aspect), an autism-specific, not-for profit organisation invited their allied health practitioners and service users including individuals on the autism spectrum and/or their families engaged with teletherapy services to complete a voluntary survey. The aim of this research was to add to the emerging discourse around teletherapy for individuals with autism, to gain point in time practice-based insights into teletherapy, both successes and barriers based on the COVID-19 pivot to teletherapy. It is anticipated these insights may contribute to the integration of teletherapy as part of service design for the broader disability sector.

## 2. Materials and Methods

### 2.1. Participants

Practitioner participants included speech pathologists, occupational therapists, psychologists, educators, and social workers from New South Wales (n = 100), Australian Capital Territory (n = 24), South Australia (n = 6), and Victoria (n = 11) at Aspect who were delivering NDIS funded teletherapy services to participants on the autism spectrum and/or their caregivers.

Service user participants included individuals on the autism spectrum and/or their caregivers who had received a teletherapy service from Aspect and were in locations across Australia. Throughout the data collection period, a total of 924 individuals on the autism spectrum and/or their caregivers received teletherapy services. Due to the anonymous nature of the survey, we were unable to extract any identifying information about service users.

### 2.2. Design

The study used a concurrent mixed methods design [28] to triangulate information from both service users and practitioners via quantitative and qualitative data.

Practitioners and service users were invited to complete an anonymous survey via Survey Monkey on their experiences of teletherapy (See Table 1).

A total of 164 practitioners were delivering teletherapy services for Aspect at the time and were invited to voluntarily complete a survey via email. Responses were collected between the 11 and 18 May 2020, approximately two months after all in person services changed to teletherapy in response to COVID-19.

Service users were invited to complete a short survey following each teletherapy session (See Table 1). The questions were designed to be a brief snapshot of service user experience of the teletherapy session during COVID-19 lockdowns. These responses were anonymous and service users could respond on multiple occasions. Data were collected from service users between 2 April and 7 September 2020. Data collection received retrospective Human Research Ethics Committee approval at the University of Sydney on 16 September 2020 (2020/457).

A total of 141 individuals completed the practitioner survey (86% response rate), and a total of 806 responses were collected from service users following teletherapy sessions. The service user response rate was unknown due to the nature of the anonymous survey and the ability for participants to respond on multiple occasions.

### 2.3. Data Analysis

Quantitative data was downloaded from survey monkey and input into SPSS (Version 24, IBM Corp., Armonk, NY, USA) [29] for analysis. Descriptive statistics were calculated for practitioners’ ratings of experience of providing teletherapy services, service users rating of satisfaction with the support provided, and service users rating of technical quality. A Pearson-product moment correlation [30] was conducted between service users scores on level of support provided and technical quality.

Qualitative data was exported and analysed using NVivo (Version 11, QSR International Pty Ltd., Melbourne, NSW, Australia) [31]. The first author conducted a thematic analysis of the data following the six steps as outlined by Braun and Clarke [32]. The author began with becoming familiar with the data before generating initial codes. The author then went on to search for themes in the codes and iteratively reviewed these themes. Finally, the first author defined and named these themes. The second author then reviewed the full data set to attain a consensus that the codes and themes accurately represented the data.

## 3. Results

The qualitative and quantitative data from both participant groups has been combined, revealing five themes which were identified during analysis; (1) technology—love it or hate it; (2) teletherapy as a “new normal”; (3) short term pain, for long term gain; (4) the shape of service delivery has changed; (5) is teletherapy always an option?

### 3.1. Technology—Love It or Hate It

A total of 782 ratings on the technical quality of the teletherapy session were collected from service users, and 777 ratings were collected on the service user perception of the level of support provided during the teletherapy session (see Table 2).

There was a strong positive correlation between technical quality and satisfaction with the level of support provided (r = 0.64, n = 772, *p* < 0.000).

Despite the above average rating for technical quality of the teletherapy sessions, the qualitative analysis identified a significant number of comments from both service users and practitioners related to technical difficulties and barriers they experienced during teletherapy sessions. The most common barrier reported by practitioners was the families having access to, and being confident in using, appropriate technology. Poor internet connection and speed were frequently reported by both service users and practitioners, as well as difficulties with connecting to software platforms, and their audio-visual quality. Both practitioners and service users reported that this impacted the success of the session and levels of engagement.

*Internet connection issues can significantly impact on rapport building and session engagement and cause overall frustration at times*.

Practitioner 63

*Technical difficulties made it difficult to stay on task and to make the most of this session*.

Service User response 59

### 3.2. Teletherapy as a “New Normal”

Despite the qualitative reports of technical difficulties, practitioners also reported a significant amount of positive feedback from participants and their families. Some families indicated that teletherapy was more engaging and just as effective for their child as in-person services. Practitioners reported that some clients indicated that they were more comfortable with the online platform and found it less confronting than in-person therapy.

*Some of my clients are anxious about meeting new people/having people come into their home, so teletherapy is less invasive for them*.

Practitioner 46

Many families valued the continuity of service they were able to receive throughout the pandemic and expressed interest in continuing to receive service online in the future.

*Would be super useful to have the teletherapy option even post the restrictions—I find it so much easier to integrate into my work schedule*.

Service User response 465

**Figure 1:**
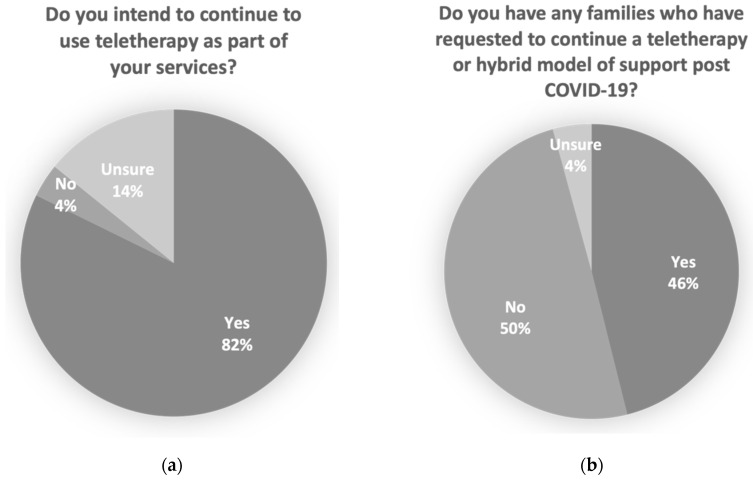
Practitioner reported intention to continue a teletherapy model of service including (**a**) percentage of practitioners who reported that they intend to continue to use teletherapy as part of their service and (**b**) percentage of therapists who have had families request to have teletherapy or a hybrid model of support continue post COVID-19.

Practitioners indicated that a majority intended to continue to use teletherapy as part of their services, and that nearly half of the families on their caseload had requested to continue a teletherapy or hybrid model of support post COVID-19 (See Figure 1).

### 3.3. Short Term Pain, for Long Term Gain

The majority of practitioners were positive about their experience of providing teletherapy services with both the mode and median rating being 4 out of 5. For practitioners, the reduction in travel was identified as one of the most significant benefits of moving to a teletherapy model of service. This was reported to have had a positive impact on practitioner productivity and the ability to see more clients on the waitlist including those in rural and remote areas. Teletherapy also resulted in service users spending less on travel, therefore releasing more funding in their budget to spend on therapy sessions.

*Potential for more efficiency by reducing travel time (which could sometimes account for 3+ hours of my day)*.

Practitioner 30

*More hours of clients plans being dedicated to therapy rather than travel*.

Practitioner 50

At the same time, there was a significant increase in planning and preparation time as practitioners learned to navigate the online space for their therapy practice. This planning and preparation time had not been anticipated, was not included in the participant’s service agreement and therefore was mostly unbillable during this time.

*It takes a significant amount of additional unbillable time per session for me to source and/or come up with appropriate and engaging activities to make Teletherapy sessions interactive and fun for my clients*.

Practitioner 29

### 3.4. The Shape of Service Delivery Changed

While practitioners reported that some clients identified as being more comfortable with the online platform and found it less confronting than face to face therapy, many practitioners reported difficulties engaging their clients, particularly young children, through the screen.

*It has been more challenging for early intervention clients as parents sometimes have different expectations (e.g., for them to sit in front of the computer and for the therapist to do 1:1 therapy with the child)*.

Practitioner 95

*It’s new and we will need to work together to find new things to make this work. It’s a challenge doing therapy this way*.

Service User response 183

This, however, had the indirect benefit of increasing parent involvement and a greater uptake of a capacity building coaching approach. Practitioners reported the benefit of higher levels of engagement with parents and a greater ability for parents to become heavily involved in therapy sessions and intervention goals in the child’s natural environment.

*Parents are becoming more confident and even providing their own strategies on therapy interventions based on their increased involvement*.

Practitioner 127

### 3.5. Is Teletherapy Always an Option?

Barriers for access to teletherapy reported by practitioners included having English as a second language, families with very little access to technology and/or high-quality internet, and families navigating through the pandemic juggling the added pressures of home schooling and working from home.

*Minus one star was due to working out logistics with families who were stressed or didn’t have adequate technology for videocall*.

Practitioner 75

*Most of my families with high needs children have opted not to engage as they did not feel they had the capacity to coordinate this as well as “life”*.

Practitioner 29

*Not all clients are suited to tele therapy—those with English as a second language, those who do not have access to technology, those with significant mental health issues or those with intellectual disabilities who find it hard how to access tele services if they don’t have a support person living with them*.

Practitioner 134

The shift to delivering services via telepractice was also reported to be a positive for practitioners where other dimensions of complexity were present. This included safety risks for the staff member due to exposure to COVID-19 or where behaviours of concern were present.

*Can continue to provide sessions if someone in the household is unwell without putting ourselves at risk*.

Practitioner 125

*No risk for therapist supporting complex PBS (Positive Behaviour Support) caseload*.

Practitioner 79

Practitioners were less convinced about the suitability for teletherapy for goals that relied heavily on observation, prompting and modelling e.g., Alternative and Augmentative Communication (AAC), social skills group and peer mediated play, and physical skills such as tying shoelaces. In these instances, practitioners perceived a need for physical presence to develop and implement strategies. Additionally, practitioners supporting individuals with behaviours of concern reported barriers in being able to adequately observe the participant behaviours in their natural environment.

*Many OT (Occupational Therapy) goals are not as effective over teletherapy such as dressing, ADL’s, motor skills as it is modelling and observation of these skills which makes therapy most effective, which is very hard to do over a camera*.

Practitioner 93

*Trialling AAC is more difficult (esp on an iPad)*.

Practitioner 113

## 4. Discussion

While teletherapy is far from a “one size fits all” approach, as reported by Pellicano, et al. [26] in their study of the experiences of individuals on the autism spectrum during COVID-19, this study adds insights from a large group of service users who valued the continuity of service they were able to receive. In addition, a significant proportion of practitioners reported that they have families that have requested to continue a teletherapy or hybrid model of service delivery post-COVID. Three quarters of practitioners in our sample have expressed an interest in continuing to use teletherapy as a part of their service delivery model post-COVID.

Therefore, the rapid upskilling and shift to online may be seen as a positive for these practitioners who have added teletherapy to their service delivery skill set. While training and support will be a constant need as technology continues to evolve and we learn more about a teletherapy model of practice, the experiences of this group of service providers and service users indicates an ongoing place for teletherapy for individuals on the autism spectrum. This is in line with the longstanding recommendations of the potential of teletherapy as a means of increasing access to therapy services into rural and remote areas [33,34]. Overall, these findings identify teletherapy as part of the “new normal”. Telepractice literature has primarily focussed on the feasibility and acceptability of a teletherapy approach, often in comparison to in-person supports. Second generation teletherapy research is required to explore the efficacy of a hybrid approach, which may represent increased efficiencies in current service design [35]. The hybrid model has already shown promise in improving mental health outcomes in rural areas [36] and support for caregivers of children with a diagnosis of attention deficit hyperactivity disorder [37]. In their commentary on post-pandemic hybrid approach to service delivery in India, Westwood [35] suggests the challenges that remain include digital education, the integration of technology into current care pathways, and creating seamless systems.

Advances in hardware, software, and Internet speed have greatly improved the technical reliability, scalability and quality of teletherapy services [38]. While ratings for both technology and support provided were above average, the strong positive correlation between the two would suggest that efficient access to and use of technology for teletherapy may impact the perceived level of support that is provided from service user perspectives. This finding aligns with broader research that has indicated that technology has not been a barrier for successful outcomes [15]. While the current study cannot correlate technical issues with outcomes, and represents the views of a narrow participant group, the results do suggest that initial investment in reliable technology needs to be made by both practitioners and service users to work towards a successful teletherapy service. For practitioners, this may mean ensuring they have access to a stable platform, reliable internet connections and training to trouble shoot any technical issues. For service users, there may be a need for training and ongoing support from the service providers admin support team to navigate the shift to teletherapy prior to beginning a teletherapy service with the practitioner. Consideration of the internet access available to service users will influence choices about teletherapy options and potential locations for access in community locations such as schools and libraries. Service users and service providers can engage in these conversations as part of establishing service agreements to ensure shared expectations are developed and any infrastructure barriers are addressed [24].

Video conferencing was the primary mode of service delivery offered for Aspect staff and clients, the participants in this research, and may be the preferred mode of teletherapy delivery, but this was not explored as a part of this research study. However, other studies have recommended offering multiple modalities, such as telephone calls, emails, and/or text messages to improve outcomes and engagement [24]. Web-based apps and resources such as those found on Boom Learning™ and Everyday Speech™ may further support the engagement in teletherapy sessions. McCrae et al. [39] found in their study of cognitive behavioural treatment for childhood insomnia in children on the autism spectrum and their parents, that practitioners used email to connect with families between sessions and that almost half of families suggested a “booster call” as an addition to treatment. Similarly, Lerman et al. [25] trained staff to adopt and implement telepractice observation via multiple modalities, for example, via audio and video recordings, and reported this may go some way to increasing the practitioner’s ability and confidence in observing the participant.

Reduction in travel has been consistently reported as one of the major benefits of moving to a teletherapy model of service, with more time being able to be spent in direct support [7,16,40]. While the current study supported these findings and their impact on increasing therapy hours, we also discovered that the rapid shift to teletherapy also resulted in a significant amount of time spent on planning and preparation for teletherapy sessions. Due to the rapid shift to online service, these hours were unexpected and therefore unbillable under the NDIS service agreements [24]. Future planning when developing service agreements may need to account for preparation time when establishing a teletherapy service as a way to allow practitioners to adapt and individualise best practice autism support for the online environment.

As reported by Johnsson et al. [16], this preparation time may reduce as practitioner’s increase in confidence and competency to adapt their practice for the online environment. Our findings are reflective of the pandemic context, which required a rapid shift to teletherapy, and did not allow for the upskilling of practitioners, or preparation of service users prior to this substantial shift to online service delivery. This finding suggests that practitioners should be given time to undergo ongoing teletherapy training and support in order to continue to adapt their model of practice. This may include discipline specific modules on adapted practice, practical support for resource adaptation, video and role play sessions, online observation sessions, and joint sessions. Due to the rapidly evolving nature of teletherapy, this may need to be updated regularly to stay abreast of teletherapy developments and advances in technology.

Consistent with Wallisch et al. [22], practitioners reported that one of the direct benefits of delivering services via teletherapy to children on the autism spectrum was the increase in parent involvement and capacity to implement support within the individuals’ everyday environments. However, we did find that some practitioners had difficulties adjusting to this new way of delivering supports. Lawford, et al. [41] indicated a need for practitioner development and supports to assist with decision making and adapting their practice. While the practitioners in the Wallisch, et al. [22] study were given training and support to carry out their intervention, participants in this study were not afforded this opportunity in the 2020 COVID-19 context of a rapid change to teletherapy service delivery. Similar to previous recommendations [24], we found that telepractice relies heavily on a coaching model of practice and therefore, innovation and effective training is indicated for staff to shift to a coaching approach that supports families in implementing family centred goals and strategies.

At the onset of the pandemic when all in person services were suddenly replaced by teletherapy, it was to be expected that some clients and families would experience difficulty with this shift. While the shift was reported to be positive by many in being able to lower the health risks for all and continue to provide services for families, we found that some practitioner reported barriers with supporting fine motor skills due to limitations on the ability to observe, prompt and model. This is consistent with previous research [16], however, telepractice is still an emerging model of delivering therapy supports, and techniques for increasing observation, prompting, and modelling may be developed using portable cameras and a more agile approach to therapy sessions as practitioners navigate the possibilities of a teletherapy context.

There are, however, still many questions left unanswered about whether teletherapy is always an option. For example, there is no research to date on the role of interpreters in a teletherapy service and their impact on access and outcomes for individuals on the autism spectrum and their support teams who are receiving services in their second language. During this time of unprecedented change, there has been a rapid upskilling of practitioners in adapting in person services for the online environment. There is a need for further targeted research to identify and mitigate barriers reported in delivering a teletherapy service.

The brief set of questions asked of service users and practitioners were intentionally broad to allow for them to speak of their experience of the rapid shift to teletherapy as a model of practice. Limitations, however, should be noted in that while the results in this study may be applied to the broader disability sector, the sample is not representative of the autism service delivery sector, and other organisations and service users may differ in their experiences. Due to the anonymity of the surveys and their brevity, the results are unable to be discussed in relation to specific practitioner disciplines (e.g., speech pathologist, occupational therapist, psychologist etc.), or the differing perspectives provided by an individual on the autism spectrum or their caregiver. The perspective of service users who chose to decline teletherapy services was also out of the scope of the current study. Online fatigue related to COVID-19 lockdown orders, and the subsequent transition of all services online may have also played a role in the findings from this study. Therefore, the findings from this brief point-in-time study should be applied as a starting point at a service wide level for further exploration in a specific context. Further research is needed to confirm these results in the broader sector, to understand the unique experiences across allied health disciplines, and to investigate the differences for individuals on the autism spectrum receiving teletherapy with that of their caregivers.

## 5. Conclusions

This study contributes to the discourse in understanding adaptations and gaps in practice in moving to an online model, the barriers to effective service provision, including technology, family complexities and specific goals, as well as the shift in practice models. While COVID-19 has brought forward incredible advances in telehealth models of practice in a short period of time, we have also learnt a considerable amount about issues in digital accessibility [42]. What’s needed now is to take stock and understand what we have learnt in terms of what has worked, for who, and in which context. To look towards the future of a post pandemic hybrid approach, we need to delve more into the experiences of a broad range of individuals on the autism spectrum, their caregivers, and their local support teams. This will help to address and potentially break down the barriers we have seen in this preliminary study to create a sustainable model of service delivery that augments existing in person services for a variety of individuals with diverse needs. We also need to harness the high level of innovation that has taken place within this timeframe to develop practice guidelines and training for new graduates and practitioners interested in incorporating this model of service delivery as part of the suite of options for NDIS participants.

## Figures and Tables

**Table 1:** Survey Questions.

**Practitioner Survey Questions**
Please rate your experience of providing teletherapy services (Likert scale 1–5) Comment on why you gave the above rating (Comment box) What are the benefits of delivering a teletherapy service? (Comment box) What are the challenges of delivering a teletherapy service? (Comment box) Do you intend to continue to use teletherapy as part of your services? (Yes, no, unsure, comment box) Do you have any families who have requested to continue a teletherapy or hybrid model of support post COVID-19? (Yes, no, unsure, comment box)
**Service User Survey Questions**
Can you please rate the technical quality of your [platform] teletherapy session? (Likert scale 1–5) Can you please rate your satisfaction with the support provided during your teletherapy session? (Likert scale 1–5) Any comments? (Comment box)

**Table 2:** Service user ratings following teletherapy sessions.

Ratings	Average Rating (Scale 1–5)
Service users rating of satisfaction with the support provided Service users rating of technical quality	4.5 4.0

## Data Availability

All data is retained on the University of Sydney Research Data Store under password protection. It is not publicly available.
